# Breast Cancer Detection from a Urine Sample by Dog Sniffing: A Preliminary Study for the Development of a New Screening Device, and a Literature Review

**DOI:** 10.3390/biology10060517

**Published:** 2021-06-10

**Authors:** Shoko Kure, Shinya Iida, Marina Yamada, Hiroyuki Takei, Naoyuki Yamashita, Yuji Sato, Masao Miyashita

**Affiliations:** 1 Department of Integrated Diagnostic Pathology, Nippon Medical School, Tokyo 113-8602, Japan; 2 Department of Breast Oncology, Nippon Medical School, Chiba Hokusoh Hospital, Chiba 270-1694, Japan; shinya1s@nms.ac.jp; 3 Faculty of Medical Science, Nippon Sport Science University, Kanagawa 227-0033, Japan; myamada58@nittai.ac.jp; 4 Department of Breast Surgery and Oncology, Nippon Medical School Hospital, Tokyo 113-8603, Japan; takei-hiroyuki@nms.ac.jp; 5 Department of Surgery, Jizankai Medical Foundation Tsuboi Cancer Center Hospital, Fukushima 963-0197, Japan; choku@nms.ac.jp; 6 St. Sugar Canine Cancer Detection Training Center, Chiba 294-0226, Japan; power_of_dog_satoh@yahoo.co.jp; 7 Nippon Medical School, Tokyo 113-8602, Japan; miyashit@nms.ac.jp; 8 Twin Peaks Laboratory of Medicine (TPLM), Yamagata 999-4331, Japan

**Keywords:** dogs, diagnosis, canine cancer detection, breast cancer, urine sample

## Abstract

**Simple Summary:**

This study aims to assess whether the urine sample can be used for breast cancer screening by its fingerprints of volatile organic compounds using a single trained sniffer dog. A nine-year-old female Labrador Retriever was trained to identify cancer from urine samples of breast cancer patients. Urine samples from patients histologically diagnosed with primary breast cancer, those with non-breast malignant diseases, and healthy volunteers were obtained, and a double-blind test was performed. The trained dog in this study could accurately detect breast cancer from urine samples of breast cancer patients. These results indicate the feasibility of a method to detect breast cancer from urine samples using dog sniffing in the diagnosis of breast cancer.

**Abstract:**

Background: Breast cancer is a leading cause of cancer death worldwide. Several studies have demonstrated that dogs can sniff and detect cancer in the breath or urine sample of a patient. This study aims to assess whether the urine sample can be used for breast cancer screening by its fingerprints of volatile organic compounds using a single trained sniffer dog. This is a preliminary study for developing the “electronic nose” for cancer screening. Methods: A nine-year-old female Labrador Retriever was trained to identify cancer from urine samples of breast cancer patients. Urine samples from patients histologically diagnosed with primary breast cancer, those with non-breast malignant diseases, and healthy volunteers were obtained, and a double-blind test was performed. Total of 40 patients with breast cancer, 142 patients with non-breast malignant diseases, and 18 healthy volunteers were enrolled, and their urine samples were collected. Results: In 40 times out of 40 runs of a double-blind test, the trained dog could correctly identify urine samples of breast cancer patients. Sensitivity and specificity of this breast cancer detection method using dog sniffing were both 100%. Conclusions: The trained dog in this study could accurately detect breast cancer from urine samples of breast cancer patients. These results indicate the feasibility of a method to detect breast cancer from urine samples using dog sniffing in the diagnosis of breast cancer. Although the methodological standardization is still an issue to be discussed, the current result warrants further study for developing a new breast cancer screening method based on volatile organic compounds in urine samples.

## 1. Introduction

Breast cancer was considered a major health issue among women, and is the second most common cause of cancer death throughout the world [1]. Early detection of the breast cancer is important for more efficient treatment. Currently, mammography (MG) is the most commonly used screening test, and has a reported sensitivity and specificity of 77% and 91%, respectively [2]. Although breast cancers can be detected during the asymptomatic phase and reduce mortality in women of certain ages [3,4,5], MG still has several challenges. First, prevalence of MG is not sufficiently utilized even in developed countries. The rate of check-ups for women aged 65−74 years is 16−20% and 43−46% for women aged 40−54 years in Japan [6]. Second, non-malignant lesions are also detected, which sometimes leads to unnecessary testing, treatment, and anxiety [7], and at the same time, MG is less sensitive in dense breast [8]. Furthermore, mortality reduction in women ages <40 years has not yet been proven. Third, MG is associated with significant pain due to the relatively strong pressure applied to the breast. Fourth, there is the risk of radiation exposure especially in younger women with abnormal germline genes [9]. Given above drawbacks of MG, an alternative test with better compliance is needed to detect breast cancer in an early stage.

Cancer detection by dog sniffing (hereinafter referred to as “canine cancer detection”) has been one of the candidates as a new method to detect breast cancer. Detection threshold has been shown to be as low as 1.5 parts per trillion (ppt) [10]. Trained dogs can successfully discriminate between patients with cancers of skin [11,12], bladder [13], lung [14,15,16,17,18], breast [14,19,20], prostate [20,21,22], ovary [23,24,25], colorectal [19,26], liver [27], and uterine cervix [28,29] from controls on the basis of odors in breath, urine, blood, or cell culture medium. However, the canine cancer screening itself is a difficult technique to disseminate for a large population. The accumulating results, though, indicate the high potential of a new cancer screening method based on the volatile organic compounds (VOCs). So far, this attempt has not been done with urine samples of breast cancer patients. Our final goal is to develop a newly non-invasive breast cancer screening method based on the VOCs. As a first step, this study is aimed to assess the potential of urine samples for breast cancer screening using a single trained sniffer dog. In this report, we also assessed our established method according to the recent recommendation of the methodology and discussed it for future research.

## 2. Materials and Methods

### 2.1. Patients and Controls

Patients with primary breast cancer, patients with non-breast malignant diseases, and healthy control volunteers at Nippon Medical School Chiba-Hokusoh Hospital and the Jizankai Medical Foundation Tsuboi Cancer Center Hospital from January 2011 to October 2012 were enrolled. Diagnosis was based on clinical assessment using MG and/or ultrasound and confirmed preoperatively by histological examination of core needle biopsy (CNB) samples. Patients who received a surgical operation before urine sample collection, and those with other types of cancer were excluded. Patients with non-breast malignant diseases were confirmed by biopsy. For female patients, MG and/or ultrasound was performed to rule out breast cancers. Healthy volunteers were verified with systematic cancer screening tests including blood test, chest X-ray, abdominal ultrasound, MG, and gynecological examination.

### 2.2. Urine Sampling

Urine samples of the participants were collected with paper cups (Harn cup laminate A, Nissho Sangyo, Tokyo, Japan), and transferred to sterile test tubes (Sterile SP tube, Eiken Chemical Co., Tokyo, Japan) and each test tube was sealed with a cap. Urine samples of the breast cancer patients were collected a few days prior to surgery. The test-tube samples were then stored at −20 °C until 1 mL of the selected samples was used for the dog sniffing test. All the urine samples were collected in the participated two hospitals, and the samples were strictly handled and stored according to the described manner.

### 2.3. Dog and Training

**Figure 1:**
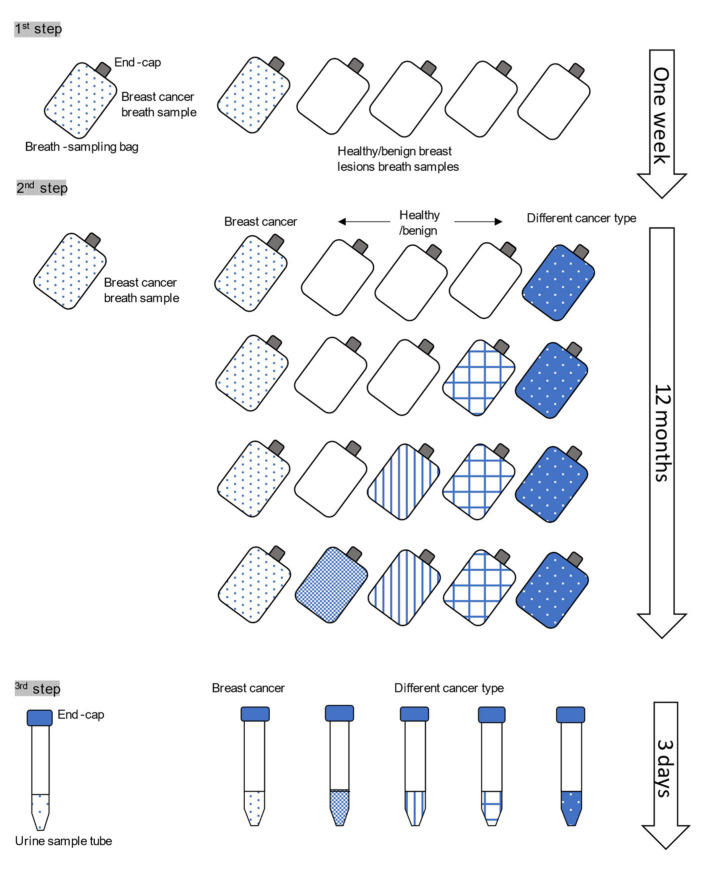
The training steps of the dog. The training consists of the three steps. In the first step, the dog was trained to detect the breath sample bag of the breast cancer patient from one breast cancer and four healthy/benign breast lesion controls. In the second step, the controls consisted of other cancer type and healthy/benign breast lesion controls. In the third step, the training was performed using the urine samples.

We previously trained a dog, who could distinguish urine samples of various types of cancer patients from those healthy and benign lesions [29]. For the present study, we trained another dog to be able to distinguish urine samples of breast cancer patients from non-breast malignant patients. Dog selection in this study was critical. A nine-year-old female Labrador Retriever was provided by the St. Sugar Canine Cancer Detection Training Center in Minamiboso City, Chiba, Japan [26,29]. The dog had passed preliminary tests confirming the ability to selectively sniff both the breath and urine of cancer patients [26]. Originally, the dog was a water rescue dog, and then, because of her high ability of sniffing out and eagerness, she was recruited to have a cancer detection training. She was trained by a professional trainer for cancer detection with a similar procedure described in the previous report [26,29]. The breath samples and urine samples used in the training steps were collected from several hundred cancer patients, five benign breast lesions patients (four fibroadenomas and one intraductal papilloma), and about 500 healthy volunteers recruited using the Internet. Briefly, our training method consisted of the following steps (Figure 1): in the first step, the dog was trained to detect a breast cancer breath sample from five breath-sampling bags with the end caps on, which included four breath samples from healthy and benign breast lesions. In the second step, the dog was trained to detect a breast cancer breath sample from five breath-sampling bags with the end caps on, which included three healthy or benign breast lesions and one non-breast malignant disease breath samples. As the dog successfully accomplished this task, the healthy controls and benign breast lesions were gradually replaced with non-breast malignant disease breath samples. Each training session was considered complete when the dog correctly detected breath samples from a cancer patient and four controls in dozens of trials, and it took about 12 months and 10 days for these steps. In the final step, the dog was trained to detect a breast cancer urine sample from five samples which included four non-breast malignant disease urine samples. The final step took three days to complete. The dog’s correct indication is sitting down in front of the target sample. Every time the correct indication is seen, the dog was rewarded and reinforced by simultaneous play with a tennis ball. In this way, the dog was trained to be able to detect breast cancer patients’ urine samples. Under certain conditions, the dog sniffing test could not be conducted because the dog could not maintain concentration. These included weather conditions such as high temperature and high humidity in summer.

### 2.4. The Testing Settings

#### 2.4.1. The Test Box

The test boxes were wooden, storage containers 27 × 30 × 20 cm in size. Each box was equipped with a 10 cm wall inside to hold a urine sample tube. Each box was covered with a metal mesh to avoid the dog’s direct contacting with the test sample.

#### 2.4.2. Detection Testing of Urine Samples from Breast Cancer Patients

**Figure 2:**
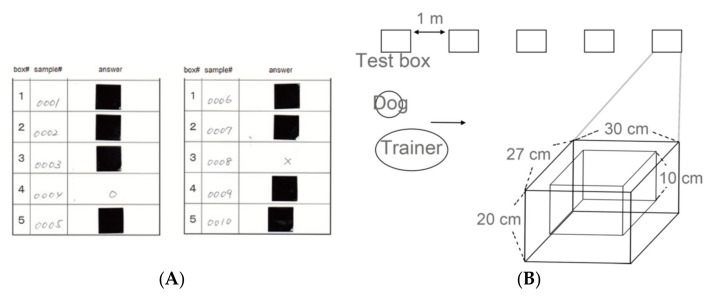
Test design. (**A**) The answer sheets used in the test. The test sample numbers and box numbers were shown on the answer sheet. The box number for the cancer sample was identified by an adjacent circle, and box numbers for the other samples were marked with an adjacent cross. The marks were then covered by a non-reattachable sticker. (**B**) The test boxes are wooden, storage containers 27 × 30 × 20 cm in size. Each box is equipped with a 10-cm deep wall inside to hold either a breath sample bag or a urine sample tube. The five test boxes were placed in a straight line on the floor at a distance of 1 m apart.

The detection testing was conducted in a similar way described in the our previous publication [29]. Test tubes containing new urine samples of breast cancer patients, as well as those of healthy controls, were used in each test. These samples were different from those used in the training. The tubes were kept separate to avoid any possibility of contamination of the control samples with potentially volatile organic compounds (VOCs) from the cancer samples. A chart to randomize numbers was used to determine the order in which the urine samples were placed in the boxes. The number was written on the sample and converted from a serial number to a test number by a third party at the same time. The test number and test box number were recorded on an answer sheet. Since the dog was to be rewarded for a correct response by playing with the tennis ball, the answer had to be known as quickly as possible by the trainer and the dog, so the answer card format shown in Figure 2A was used. On the answer sheet, the urine sample of breast cancer patients was identified by an adjacent circle (○) next to the test box number, and urine samples from the control patients were marked with an adjacent cross (x). The marks were then covered with a sticker, which once detached, could not be reattached. In each test run, one breast cancer urine sample and four control samples were used. The assistant placed the test tube samples in the boxes, which were placed in a straight lineup on the floor, one meter apart, according to the number noted on the answer sheet (Figure 2B). The samples were put out in sequence from boxes 1 to 5. The dog trainer, assistant, and experimenter did not know the positive sample. The tube was handled with care, not to be contaminated with each other. The testing was performed in a double-blinded way (i.e., the sample content was unknown to the dog, the trainer, and the assistant [30]). At the beginning of the test, after the dog had been trained to concentrate, the dog’s nose was exposed to a standard urine sample from breast cancer patient used in the training steps described above. The trainer then attached a leash and walked the dog by the test boxes to permit her to sniff the urine samples.

#### 2.4.3. Evaluation of the Dog’s Response

The dog sat in front of the positive samples after sufficiently smelling all five boxes in each test run. The dog’s indications were categorized as follows: (1) Sitting down in front of a sample box containing a urine sample from a breast cancer patient (true positive in sensitivity calculations) and (2) only sniffing a control sample and not sitting in front of it (true negative). Incorrect actions included (1) sitting in front of a control sample (false positive) and (2) not sitting in front of a sample from a breast cancer patient (false negative). The verdict of the test was determined after confirming that the dog did not move spontaneously for three seconds. If the dog started to move before that time, the test verdict was temporarily suspended. In such cases, assessment was determined when the dog sat in front of the test box and did not move for three full seconds. Once the dog indicated a sample, she was given a reward. According to the dog’s indication (described in the next paragraph), the assistant peeled off the sticker next to the box number on the answer sheet and checked the results. The answer sheets were collected by mail and checked to verify whether the test had been conducted correctly.

For each test, the dog’s concentration level (high, normal, or low) was assessed and recorded. Tests were always held on days when the dog’s concentration level was high. Tests were not conducted during extreme environmental conditions such as days with high temperature and humidity, or during irregular natural phenomena such as earthquakes and typhoons, as the dog’s concentration level at such times was low.

### 2.5. Statistical Analysis

The Kruskal–Wallis test was used to analyze the clinical characteristics of the patients and controls. The percentage of correct detection per session was calculated for each test-run. Diagnostic accuracy was calculated as the sensitivity and specificity of the dog’s identification of positive urine samples compared to the histopathological diagnosis of breast cancer. Thus, sensitivity of the test is the proportion of cancer samples correctly identified by the dog while specificity is the proportion of control samples negatively indicated by the dog. A *p* value < 0.05 was considered statistically significant. All statistical analyses were completed using SPSS v.25 (IBM Corp., Armonk, NY, USA).

### 2.6. Ethics Approval and Consent to Participate

Participants voluntarily enrolled in this study and provided written informed consent. This study was conducted in accordance with the principles embodied in the Declaration of Helsinki, and was approved by the ethics committees of Nippon Medical School Chiba Hokusoh Hospital (IRB#320).

## 3. Results

### 3.1. Patients

A total of 200 participants were randomly selected in the study, and included 40 patients with primary breast cancer, 142 patients with non-breast malignancies, and 18 healthy individuals. All participants were female, except for one male person in the healthy group. Histological diagnoses were ductal carcinoma in situ (six cases), non-specified invasive ductal carcinoma/ invasive carcinoma (33 cases), and mucinous carcinoma (one case). Pathological stages of the breast cancer patients were classified according to the Union for International Union Cancer Control (UICC) classification as follows: Stage 0 (ductal carcinoma in situ) for 6 cases, Stage I for 19 cases, Stage IIA for 13 cases, and Stage IIIB for 2 cases). One patient with invasive ductal carcinoma, Stage IIIB had preoperative chemotherapy before the operation and collecting a urine sample. Non-breast malignancy patients are listed in Table 1. Median ages of breast cancer patients, non-breast malignancy patients, and healthy individuals were 57.5 (range 28–84), 57 (range 18–88), and 52 (range 27–66) years old, respectively. Age distribution did not significantly vary among the patients and controls (*p* = 0.087).

### 3.2. Dog Condition and Round Times before Decision

A total of 40 runs were carried out. The combinations of the samples in each test-run are listed and summarized in Table 2. In 4 out of 40 times, the dog’s concentration level was low, and the remaining runs were normal. The dog’s low concentrations were noted for two days, when test-run numbers 18–22, which were the hottest days in July, were performed. On these days, the room temperature was 26.3 °C to 26.8 °C, and humidity was 83%. The round times before the dog’s response ranged from one to three times. In further detail, the dog made one round in 14 runs, two rounds in 19 runs, and three rounds in seven runs. No adverse events, injury, or illness to the dog was observed.

### 3.3. Sensitivity and Specificity of the Detection Test

Comparison of the cancer determination by dog sniffing versus pathological diagnosis among cancer patients and controls was calculated. The dog detected the breast cancer samples correctly in all test-runs (40/40). Thus, among the breast cancer patients and controls, overall sensitivity and specificity were both 100%.

## 4. Discussion

The accumulating results by some researchers using sniffer dogs have indicated the high potential of a new cancer screening method based on the volatile organic compounds (VOCs). The novelty of our current study was to investigate the feasibility of breast cancer screening using urine samples based on the VOCs sensed by the trained dog. The trained dog detected and distinguished urine samples of breast cancer patients from a control group comprising of a variety of other malignancies and healthy volunteers, and 100% sensitivity and specificity rates could be achieved in the double-blind test series. Up to now, the efficacy of urine samples has not been well clarified. Using urine samples is useful because of its simplicity and non-invasiveness. Some trained dogs were reported to discriminate between the urine of patients with urinary tract and prostate cancers from those of controls [13,21]. This is the first, preliminary study indicating the feasibility of developing a new breast cancer screening method using urine samples based on VOCs.

Originally, in 1989, the hypothesis that dog can smell a cancer odor was raised during the consultation with a woman who claimed to have sought medical help as a direct result of her dog’s inordinate interest in a skin lesion, which subsequently proved to be a malignant melanoma [31]. A similar case of patient–dog interactions leading to cancer diagnoses was subsequently reported, suggesting the possibility of the existence of a cancer-specific odor [32]. Initially, these “anecdotal” events were not supported by evidence. However, the following studies have demonstrated canine cancer detection for cancer screening is promising, feasible, and safe (Table 3) [11,12,13,14,15,16,17,18,19,20,21,22,23,24,25,26,27,28]. McCulloch et al. reported that trained dogs could successfully detect breast cancers using exhaled breath samples [14]. Breath samples from 31 breast cancer patients and healthy control patients were used, and sensitivity and specificity were 0.88 and 0.98, respectively, across all stages. Sonoda et al. further investigated the utility of canine cancer detection in CRC using breath and watery stool [26]. Sensitivity and specificity in comparison with diagnosis by colonoscopy were 0.91 to 0.97, and 0.99, respectively. In order to determine whether a specific cancer odor does exist, or a particular natural scent disappears due to the cancer, a mixture of watery stool of CRC cancer patients and controls was produced, and the sample could be correctly identified by the dog. From this, it was surmised that chemical compounds from cancer could be circulating throughout the body. Next, focus was placed on whether these odors were cancer-common or cancer-specific. In several subsequent series, when one type of cancer sample was used as the standard scent, the dog was able to differentiate between other types of cancers [26,33]. Seo et al. also reported that metabolic wastes of both breast and CRC in vitro have a common specific odor [19]. On the other hand, several types of cancers which were included as controls could be successfully identified as the targeted cancer by the sniffer dog, which is consistent with the results of this study [24]. These results suggest that there may exist common scents among various cancer types, and each cancer type has a cancer-specific odor [14,26]. We recently reported a trained dog, who was trained to detect various cancers from healthy controls or benign lesions [29]. The current dog is especially used as a breast cancer-specific odor. The dog in this test successfully differentiated breast cancer from non-breast malignancies and healthy controls, and this concurs with previous studies [24].

The present test data showed a higher sensitivity and specificity compared to other previous reports. One possible reason is the environmental settings of the test-run, which allowed the dog to respond without stress. Tests were not carried out under stressful conditions for the dog. Detection accuracy may be influenced by the condition of the dog, and therefore performance should be systematically monitored [30]. In our study, the dog’s concentration was bothered on hot and humid days. Therefore, we believe that we need training menus that allow dogs to tolerate different environments, including hot and humid weather. Local training is also a good option. Accumulated research has assessed dogs as detectors of various cancers, infectious diseases, metabolic diseases, but the inconsistency and lacking information of the training and testing the dogs make it difficult to ascertain the potential of the dog’s detection [22,30]. It is difficult to directly compare the results of experimental studies on cancer-detection dogs, because these studies vary methodically in many aspects. Recently, recommendations for the training and testing of animals on using olfaction to detect human disease were published [22,30,34]. The recommended methodologies are listed in Table 4. First, regarding the preselection of the dog, most studies used one to five dogs [14,26] without the information of pre-selection of the dogs. We recruited one Labrador Retriever. Although no clear answer exists, based on the genetic diversity of olfactory receptors, German shepherd or Labrador Retriever have good potential as sniffer dogs [33,35]. Our dog was initially recruited and trained as a water-save dog because of her eagerness and adaptability. A few years after the training, she was chosen as a sniffer dog due to her high ability to sniff objects and respond to commands. The training period varies considerably from study to study, and information is unavailable in some studies. In our study, it took about one year for the detection training. We previously trained a cancer-detection dog, which detects various cancers [29] and for the current study, we trained the dog specifically to detect breast cancer. These training were performed one by one. No previous data are available regarding how long the trained cancer-detecting ability can last. Our dog’s ability lasted during the tests for one year, and ideally, ongoing training, which cannot be discriminated from the test or operation, should be performed. Sampling urine is another issue in the experiment [20]. Sampling tubes should be simple and handy to be used by sample donors without training. In this regard, we selected ordinary sampling tubes, which can be handled without special training, but the quality is guaranteed. The timing of the sampling is important. In our study, urine samples were collected before the diagnosis, and therefore, the positive result was not due to the manipulation/biopsy of the breast, medication, or emotional stress. In addition, all the urine samples were collected in the participating two hospitals, in which situation the possibility of the confounder of the hospital odor is excluded. The samples were strictly handled and stored in the described manner. The sample storage time is not standardized, but one study described that samples stored for one to 60 months were used. In our study, samples stored for up to one year in −20 °C were applied. The sample storage at −20 °C appeared to ensure sample stability, and freeze-thaw cycles did not affect the sample quality [36]. The urine storage is especially beneficial when the patients live far away from the testing dog. In addition, it is expected that urine results may serve as an ancillary diagnosis when there is difficulty in diagnosis based on imaging and other clinical findings in early lesions. The control samples should be comparable to positive samples except for disease status. However, due to the limited numbers of the samples, we were not able to perfectly match the control samples. For future training and experiment, a “sample bank” of various materials, including more male patients, benign breast lesions, healthy people as well as post-operative and/or post neoadjuvant therapy patients, with background information is suggested. In the training setting, intermittent reinforcement generates patterns of behavior that persist even when reinforcement is no longer forthcoming [34]. In the current study, each time the dog indicated the positive target, the dog was rewarded. However, according to the recommendation, this reinforcement shall be diminished to be intermittent, for the feature practical use. For the training and testing setup, the samples were placed in a lineup in most of the previous studies, but a few were arranged in the circle [37]. Testing of four to seven samples can be recommended, since more samples may result in a lower probability of correct indication [30]. The positive samples were used mostly one, but one study applied various target samples from one to six [37]. In our current experiment, one positive out of four control samples was arranged in the lineup, and this method is in line with the previous reports. In the current experiment, urine samples of five benign breast lesions patients’ were utilized. However, these samples were used for only training, due to the limited number. Trial with variable positive numbers and controls including benign lesions—ideally reflecting the frequency of the disease prevalence—is warranted in the next step. Testing should be done with new samples because there is a risk of memorization of particular odor of the samples used for training with poor generalization. In the current study, we applied new samples to the testing. All tests, except for the early stage of the training, should be conducted using “double-blind protocol.” Double-blind refers to the dog, the trainer, and the experimenter are all blind to the target sample to avoid “Clever -Hans” phenomenon. Our study has met this recommendation.

Evidence has shown that human body emits a wide array of volatile organic compounds (VOCs), both odorous and non-odorous, depending on the individual background [38]. These VOCs are emitted throughout the body, including breath, blood, and urine [39,40]. According to analysis of VOCs, different volatile patterns have been correlated with a variety of diseases including cancers [14,38,41,42], which dogs can be trained to detect. Consequently, analysis of cancer specific VOCs is considered feasible. Some studies have attempted to demonstrate cancer-specific VOCs by utilizing gas chromatography-mass spectrometry (GCMS) [43]. The potential of VOCs in urine, breath, and blood samples to be biomarkers for an array of diseases could be demonstrated [40,44]. However, VOCs are affected by physiological factors such as dietary and smoking habits, infections, and benign diseases [45], which GCMS cannot detect all or even nearly all chemicals present [14], nor clarify the exact chemical compounds and/or their combinations. Combining this dog-based study with instrument-based, “electronic nose,” research would be mutually beneficial for further analysis [46]. Our dog-based method itself is difficult for disseminating in clinical practice, but as a preliminary result, it warrants further research developing a new breast cancer screening method based on VOCs.

This study has limitations. Our cancer detection system relies on one trained dog. An effective training protocol is essential for good performance [20], and expanding established training methods to multiple training centers with expert trainers, multiple dogs, and over several years is desired. Once the methodology and workflow are established, the cost of training is almost the same as for any other type of dog training, and is not particularly high for cancer detection dogs; the more widely used cancer-detection dogs become, the more cost-effective it will be. We hope that the public will become more aware of the cancer detection dogs and support their practical use. Second, our testing included relatively limited numbers of samples. For future ongoing experiments, a wide variety of samples for training is preferred. As a solution, an organized sample bank and training center would be helpful to expand the experiment. In addition, it would also be practical if urine tests could be performed to monitor patients after chemotherapy or surgery. In this regard, it is preferable to conduct training in a variety of situational settings close to the clinical setting. These accumulating results may let us step forward to detect the cancer-specific VOCs for development of the “electronic nose”.

The extrapolation of our results to widespread implementation is still uncertain. However, even if few dogs could be trained to detect breast cancer, the result may open the door to a robust and inexpensive way to detect breast cancer in a long-term perspective, which is the big advantage and prospective of our experiment. Dog cancer detection is entirely non-invasive, safe, and easy for both patients and everyone. The sampling and storage require no special conditions, and the samples can be stored up to several months after sampling is a great advantage, because it is not always possible to test samples shortly after the sample collection. This method would have good prospects, especially in low-income countries where common access to MG is still an obstacle. Only developed countries can adopt high technological cancer screening. In the low- and middle-income countries, despite that the efforts for cancer screening programs have been made, it is difficult to implement technologically advanced approaches. In these countries, the lack of hospitals near rural locations, the prohibitive cost of medical care, insufficient equipment, and a shortage of medical workers are obstacles to cancer screening. In addition, screening protocols in low-resource areas tend to differ from those in developed countries, i.e., for cervical cancer, visual inspection with acetic acid is a more realistic choice rather than expensive smear tests [47]. Likewise, we believe some well-trained sniffing dogs traveling around medically underserved all over the world could save many lives. Even when “a healthy control” was indicated by a trained dog, there would be a suspicion of undiagnosed/early stage cancer, and the person would be advised to undergo medical screening.

## 5. Conclusions

In conclusion, this study represents the feasibility of breast cancer screening using urine samples based on the VOCs sensed by a trained dog. Further research developing the new, electronic nose is warranted.

## Figures and Tables

**Table 1:** Number of non-breast malignancy cases and healthy volunteers used as controls.

Diagnosis	Number
Gastric cancer	38 (26.0%)
Cervical cancer	36 (24.7%)
HSIL	21 (14.4%)
Endometrial cancer	17 (12%)
Ovarian cancer	16 (11.0%)
Colorectal cancer	7 (4.8%)
Peritoneal cancer	3 (2.0%)
Uterine sarcoma	2 (1.4%)
Esophageal cancer	1 (0.7%)
Endometrial stromal sarcoma	1 (0.7%)
Vulvar cancer	1(0.7%)
Liposarcoma	1 (0.7%)
Metastatic adrenal carcinoma	1 (0.7%)
LSIL	1 (0.7%)
Total	146

HSIL, high-grade squamous intraepithelial lesion; LSIL, low-grade squamous intraepithelial lesion.

**Table 2:** Sample combinations of each test-run. Diagnosis/healthy, age, and stage are listed. Stages indicate pathological stage according to UICC classification.

	Box 1			Box 2			Box 3			Box 4			Box 5		
Test -Run	Diagnosis	Age	Stage	Diagnosis	Age	Stage	Diagnosis	Age	Stage	Diagnosis	Age	Stage	Diagnosis	Age	Stage
1	HSIL	31	NA	GC	41	IB	HSIL	36	NA	BC	33	0	HSIL	35	NA
2	EmC	71	IB	GC	77	IA	BC	74	I	GC	70	IA	GC	70	IA
3	GC	60	IB	CC	51	IIB	CC	53	IIB	CRC	63	II	BC	56	IIA
4	CC	85	IIIB	CRC	71	IIIB	CRC	68	IIIB	BC	64	IIIB	EmC	57	IIIA
5	GC	74	IB	EC	59	IA	HSIL	50	NA	HSIL	47	NA	BC	60	0
6	CRC	70	I	EmC	70	IIIC	BC	75	I	Metastatic adrenal cancer	80	IV	CC	85	IIIB
7	OC	51	IIIC	GC	51	IA	BC	49	IIA	HSIL	46	NA	EmC	44	IB
8	GC	57	IA	OC(rec)	58	NA	PerC	61	IIIC	GC	60	IIA	BC	62	IIIB
9	HSIL	26	NA	HSIL	27	NA	Liposarcoma	39	NA	BC	28	I	GC	41	IB
10	HSIL	44	NA	US	41	IV	GC	57	IIA	CC	54	IIIB	BC	50	0
11	HSIL	44	NA	ESS	41	IV	GC	57	IIA	CC	54	IIIB	BC	50	0
12	EmC	70	IB	GC	71	IA	CC	74	IB	BC	69	I	CC(rec)	69	NA
13	HSIL	30	NA	HSIL	36	NA	BC	42	I	GC	55	IV	CC	34	IA
14	CC	61	IB	OC (rec)	65	NA	BC	56	I	EmC	79	IV	GC	75	IA
15	GC	64	IA	BC	59	I	CC	55	IVB	CC	57	IA	PerC (rec)	65	NA
16	HSIL	39	NA	LSIL	44	NA	OC	61	IA	BC	47	IIA	HSIL	39	NA
17	HSIL	18	NA	CC	82	IIIB	BC	38	IIIA	CC	36	IA	HSIL	38	NA
18	HSIL	44	NA	BC	47	IIA	HSIL	47	NA	CC	41	IA	CC	44	IIIB
19	OC(rec)	56	NA	CC	57	IA	OC	61	IA	CC	61	IB	BC	59	I
20	BC	67	IIA	CC	65	IV	CC	75	IVB	CC	62	IB	GC	63	IA
21	Vulvar cancer	78	I	CC	85	IIIB	EmC	82	IV	BC	84	I	GC	77	IA
22	healthy	39	NA	EmC	49	IB	BC	48	I	GC	46	IA	HSIL	42	NA
23	GC	64	IA	BC	64	I	CC	62	IB	Healthy	27	NA	OC	61	IA
24	Healthy	35	NA	GC	41	IB	OC	45	IIIC	CC	38	IIIB	BC	46	I
25	OC	50	IC	Healthy	39	NA	GC	51	IA	BC	48	I	GC	55	IA
26	Healthy	78	NA	CC	34	IA	HSIL	33	NA	BC	34	I	HSIL	35	NA
27	Healthy	56	NA	GC	77	IA	BC	67	IIA	GC	71	IA	CRC	70	IV
28	CC	51	IIb	GC	51	IA	CC	47	CIS	BC	49	IIA	EmC	49	IB
29	GC	70	IA	EmC	70	IIIc	Healthy	57	NA	CRC	68	IIIB	BC	69	IIA
30	CC	44	IIIB	Healthy	41	NA	Uterine sarcoma	41	NA	BC	44	IIA	CC	42	IIIb
31	EmC	71	IB	BC	71	IIA	OC	88	IV	Healthy	66	NA	OC	85	IIIC
32	Healthy	51	NA	EmC	70	IB	BC	69	I	GC	70	IA	EmC	63	IC
33	CC	62	IB	OC	61	IA	Healthy	53	NA	BC	61	I	GC	62	IA
34	Healthy	50	NA	BC	56	I	OC(rec)	56	NA	GC	55	IA	CC	57	IA
35	GC	77	IA	Healthy	60	NA	EmC	77	IC	GC	75	IA	BC	75	I
36	BC	59	I	CC	61	IB	Healthy	50	NA	EmC(rec)	58	NA	GC	57	IA
37	GC	57	IIA	Healthy	57	NA	CC	53	IIB	CC	51	IIB	BC	56	IIA
38	Healthy	56	NA	CRC	63	II	BC	64	IIA	GC	60	IIA	CC	53	IIA
39	Healthy	45	NA	OC	49	IA	GC	41	IB	BC	45	I	EmC	49	IB
40	GC	64	IA	OC	68	IA	BC	64	I	OC	56	IA	Healthy	60	NA

HSIL, high-grade squamous intraepithelial lesion; NA, not applicable; GC, gastric cancer; BC, breast cancer; EmC, endometrial cancer; CC, cervical cancer; CRC, colorectal cancer; EC, esophageal cancer; (rec), recurrence; PerC, peritoneal cancer; US, uterine sarcoma; ESS, endometrial stromal carcinoma; OC, ovarian cancer; LSIL, low-grade squamous intraepithelial lesion; rec, recurrence; US, uterine sarcoma.

**Table 3:** Published reports on canine detection of various cancer types.

Reference	Cancer Type	Material	Numbers of the Tested Cases	Sensitivity	Specificity
Pickel, D.P., 2004 [11]	Malignant melanoma	tumor	7	82%	100%
Willis, 2004 [13]	bladder cancer	urine	36 108 cancer negative	41%	ND
McCulloch, M., 2006 [14]	lung cancer (LC), breast cancer (BC)	breath	LC: 55 BC: 31 83 healthy	LC: 99%	LC: 99%
				BC: 88%	BC: 98%
Gordon, R.T., 2008 [20]	BC, prostate cancer (PC)	breath	BC: 18 PC: 33	ND “no better than chance”	ND
Horvath, G., 2008 [23]	ovarian cancer (OC)	tumor tissue	31 control(fat/muscle/normal ovary)	100%,	97.50%
Horvath, G., 2010 [24]	OC	tumor tissue (T), blood (Bl)	40 controls (4 uterine corpus cancer, 2 uterine cervical cancer, 2 vulvar cancer, and healthy)	T: 100%, Bl: 100%	T: 95% Bl: 98%
Cornu, J.N., 2010 [21]	OC	urine	33	91%	91%
			33 healthy		
Ehmann, R., 2012 [15]	LC	breath	60, 110 healthy/50 COPD	71%	93%
Sonoda, H., 2011 [26]	CRC	breath (Br), stool (Stl)	Br: 33/132 healthy Stl: 37/148 healthy	Br: 91% Stl: 97%	Br:99% Stl: 99%
Horvath, G., 2013 [25]	OC	blood	42 210 healthy	97%	99–100%
Elliker, K.R., 2014 [22]	PC	urine	16 48 controls (healthy/hyperplasia)	13–25%	71%
Schallschmidt, K., 2015 [16]	LC	head space gas of cell culture		10–20%	40–50%
Hackner, K., 2016 [17]	LC	breath	29 93 without LC	Positive predictive values 30.9% Negative predictive value 84.0%	
Kitiyakara, T., 2017 [27]	HCC	breath	37 healthy	78%	ND
Guerrero-Flores, H., CC, 2017 [28]	CC	smear	50 30 healthy controls	92.80%	99.10%
Seo, I.S., 2018 [19]	BC + CRC	cell culture liquid		>90%	<90%
Junqueira, H., 2019 [18]	NCSLC	blood serum	ND healthy	96.70%	97.50%

ND, not determined; lung cancer, LC; hepatocellular carcinoma; BC, breast cancer; PC, prostate cancer: OC, ovarian cancer; HCC, hepatocellular carcinoma; CC, cervical cancer; CRC, colorectal cancer, NCCLC, non-small cell lung cancer.

**Table 4:** Recommended conditions.

Checkpoints	Methodological Recommendations	
Dogs	Breed	German Shepherd, Labrador Retriever.
Samples	Sampling tube	It should be simple and handy to be used by sample donors without training.
	Storage time	Not determined.
	Sample collection	Collection in the same location. A large numbers/varieties of the samples.
	Control samples	They are comparable to positive samples except for disease status
Training conditions	Reinforcement/ reward	Intermittent reinforcements
	Sample arrangement	Odor line-up/circle
	Positive/Negative ratio	It should reflect the disease prevalence. Positive sample prevalence reflecting the prevalence of disease in operating setting.
Testing conditions		Sample sources different from source used in training should be used.
		The dog, trainer, and experimenter are blind to the status of all samples (“Double-blinded”).
		Accurate knowledge of sample status.
		Sufficient large number of sample sources
Operation conditions		Ongoing training should be performed. Training conditions cannot be discriminable from operational conditions.
		Regular evaluation of performance with another diagnostic tool.

## Data Availability

The data presented in this study are available on request from the corresponding author.
